# Downregulation of APOBEC3G by xenotropic murine leukemia-virus related virus (XMRV) in prostate cancer cells

**DOI:** 10.1186/1743-422X-8-531

**Published:** 2011-12-12

**Authors:** Abhinav Dey, Chinmay Kumar Mantri, Jui Pandhare-Dash, Bindong Liu, Siddharth Pratap, Chandravanu Dash

**Affiliations:** 1 From The Laboratory of Retrovirology and Epigenetics, Center For AIDS Health Disparities Research, Vanderbilt-Meharry Center For AIDS Research (CFAR), Department of Biochemistry and Cancer Biology, 1050 Dr. DB Todd Jr. Blvd, Old Hospital Building, Room 5027, Nashville TN 37208, TN, USA; 2 Mass Spectrometry Core, Meharry Medical College School of Medicine, Nashville, TN, USA

**Keywords:** XMRV, APOBEC3G, Retrovirus, Prostate

## Abstract

**Background:**

Xenotropic murine leukemia virus (MLV)-related virus (XMRV) is a gammaretrovirus that was discovered in prostate cancer tissues. Recently, it has been proposed that XMRV is a laboratory contaminant and may have originated via a rare recombination event. Host restriction factor APOBEC3G (A3G) has been reported to severely restrict XMRV replication in human peripheral blood mononuclear cells. Interestingly, XMRV infects and replicates efficiently in prostate cancer cells of epithelial origin. It has been proposed that due to lack off or very low levels of A3G protein XMRV is able to productively replicate in these cells.

**Findings:**

This report builds on and challenges the published data on the absence of A3G protein in prostate epithelial cells lines. We demonstrate the presence of A3G in prostate epithelial cell lines (LNCaP and DU145) by western blot and mass spectrometry. We believe the discrepancy in A3G detection is may be due to selection and sensitivity of A3G antibodies employed in the prior studies. Our results also indicate that XMRV produced from A3G expressing LNCaP cells can infect and replicate in target cells. Most importantly our data reveal downregulation of A3G in XMRV infected LNCaP and DU145 cells.

**Conclusions:**

We propose that XMRV replicates efficiently in prostate epithelial cells by downregulating A3G expression. Given that XMRV lacks accessory proteins such as HIV-1 Vif that are known to counteract A3G function in human cells, our data suggest a novel mechanism by which retroviruses can counteract the antiviral effects of A3G proteins.

## Findings

Xenotropic murine leukemia-virus related virus (XMRV) is a member of the gammaretrovirus family that was first detected in human prostate tumors [1]. Although initial studies supported the presence of XMRV in prostate cancer tissues [2-4], since then several laboratories have failed to detect the virus in cohorts of prostate cancer patients [5-9]. Very recently, Paprotka et al. (2011) have challenged an association of XMRV with human diseases [10]. These authors have reported that XMRV may have originated by a rare recombination event during tumor passaging in mice. Therefore, it has been proposed that XMRV is a laboratory contaminant and not a human pathogen. Nevertheless, being a newly discovered gammaretrovirus and having the ability to infect human cells, XMRV may serve as a model gammaretrovirus to further our understanding of retroviral biology.

Apolipoprotein B mRNA-editing enzyme catalytic polypeptide-like 3 (APOBEC3) proteins, APOBEC3A to APOBEC3G are a class of cytidine deaminases that has been reported to restrict retroviral replication in humans [11]. In case of HIV-1, the restriction by APOBEC3G (A3G) and APOBEC3F (A3F) are counteracted by Vif that degrades A3 proteins via proteosomal degradation [12]. It has been reported that XMRV replication can be inhibited by A3 proteins such as A3G, A3B, A3F, and murine APOBEC3 (mA3) [13-17]. Although hypermutation of XMRV genome in A3G/A3F-expressing peripheral blood mononuclear cells (PBMCs) severely restrict XMRV replication in human blood, infected PBMCs have been suggested to serve as source of infectious XMRV [17]. Given that XMRV is a simple retrovirus and does not encode accessory proteins that are known to counteract A3G/A3F, these observations suggest XMRV cannot survive the restriction of innate immunity for productive infection in humans.

XMRV replicates efficiently in prostate epithelial cell lines specifically in LNCaP cells [3,18]. In addition, the prostate cancer cell line 22Rv1 has been shown to be chronically infected with XMRV and produces highly infectious virus [19]. Since host restriction factor A3G is able to restrict XMRV, the question is how XMRV replicates efficiently in these human prostate cell lines. There are at least three studies that have suggested that XMRV efficiently replicates in prostate epithelial cancer cell lines since these cells lack or express undetectable levels of A3G [13-16]. In this report, we demonstrate that prostate epithelial cell lines LNCaP and DU-145 express detectable levels of A3G by western blot analysis. We confirm the presence of A3G in LNCaP cells by mass spectrometry. We believe the results described in earlier reports on the absence of A3G in these cells may be due to the sensitivity of antibody used in their western blot analysis.

**Figure 1:**
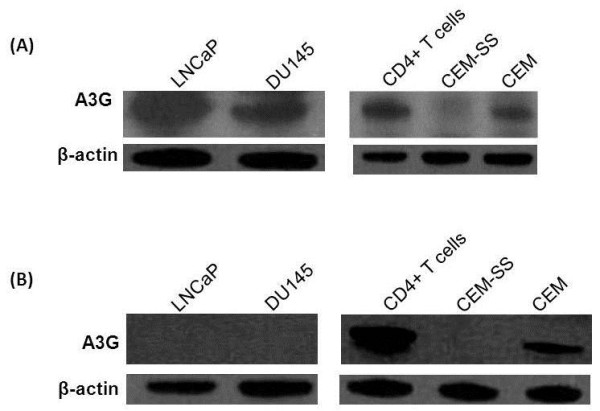
**Detection of A3G in prostate cancer cells of epithelial origin A3G was detected using two different polyclonal rabbit sera obtained from the NIH AIDS Reagent Program**. Anti-ApoC17 was raised against a synthetic peptide comprising of the 17 C-terminal residues of A3G (Cat. No. 10082), while the other was (Anti-ApoC29) Anti-A3G C-terminal antisera raised against a C-terminal peptide representing the last 29 amino acids of human A3G coupled to a hapten (Cat. No 10201). As a loading control β-actin (Sigma Co., USA) was used. For immunoblot analysis, cell lysates (5 μg) were subjected to SDS-polyacrylamide gel electrophoresis and western blot analysis with the appropriate antibodies. Western blot analysis using, (**A**) Anti-ApoC29 and (**B**) Anti-ApoC17.

**Figure 2:**
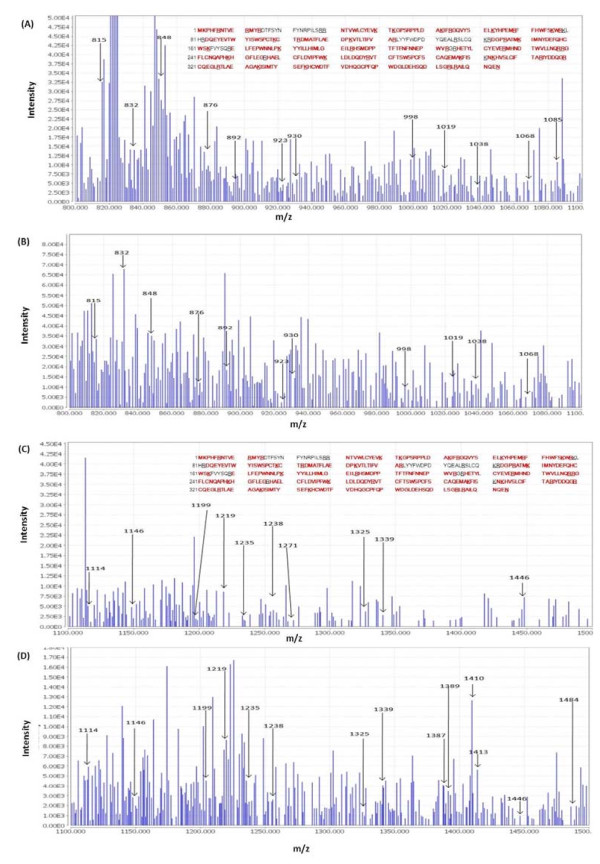
**Proteomic Analysis for detection of A3G in LNCaP cells The cell lysate from LNCaP cells was loaded onto SDS-polyacrylamide gel (4-12% gradient gel) and resolved through electrophoresis as described in Figure 1**. Pure recombinant A3G was also run on a separate gel. Both the gels were stained with Coomassie brilliant blue. One section of the gel was subjected to immunoblotting using anti-ApoC29 to assist in A3G identification in the gel for mass spectrometry. The gel bands corresponding to A3G protein were excised and were subjected to in-gel trypsin digestion based on the manufacturer's protocol (Thermo Scientific). The resulting peptides were analyzed using a Thermo Finnigan LTQ ion trap instrument ESI. Peptides were separated on a packed capillary tip (Polymicro Technologies, 100 μm × 11 cm) with Jupiter C18 resin (5 μm, 300 Å, Phenomenex) using an in-line solid-phase extraction column (100 μm × 6 cm) packed with the same C18 resin. The total ion chromatogram for the digested pure A3G and A3G from LNCaP cells were compared. The common regions of similar retention time (≈2 minutes) were analyzed to search for m/z peaks corresponding to A3G in the LNCaP sample. The representative peaks were matched with the m/z peaks corresponding to pure A3G. This search revealed that more than 85% of the sequence could be identified in the spectra in the A3G band detected in LNCaP cells. (**A**) Spectra of purified A3G (m/z 800 to 1100); (**B**) Mass spectra of A3G from LNCaP cell lysate (m/z 800 to 1100); (**C**) Spectra of purified A3G (m/z 1100 to 1500); and (**D**) Spectra of A3G from LNCaP cell lysate (m/z 1100 to 1500). The detected peptides from A3G sequence have been shown in red. The X-axis represents m/z and the Y-axis represents intensity.

We detected A3G in LNCaP and DU145 cells (Figure 1A) using a polyclonal antibody (anti-ApoC29) raised against the 29 amino acid (aa) of the C-terminal end of A3G protein (NIH AIDS Reagent Program Catalog # 10201). We used lysates of CD4+ T cells and CEM cells as positive controls and CEM-SS cells as negative control for A3G expression by western analysis (Figure 1A). These results contradict the previous reports that were unable to detect A3G in these cells [13-16]. We realized that the antibody used in the previous reports was a polyclonal antibody (anti-ApoC17) raised against the 17aa of the C-terminal of A3G protein (NIH AIDS Reagent Program Catalog # 9968). Therefore, we repeated our western analysis using anti-ApoC17 and similar to the earlier reports we could not detect A3G in LNCaP and DU-145 cells (Figure 1B). Therefore, we examined whether the band detected in Figure 1A using anti-ApoC29 contains A3G protein. To confirm this, we carried out Electrospray Mass Spectrometry (ESI) analysis by cutting out A3G band from the coommassie stained gels. After a comparative analysis of the spectra with purified A3G, we could map more than 85% of A3G peptides in the A3G band detected in the lysates of the LNCaP cells (Figure 2). Since the anti-ApoC29 used in our study has been used by several laboratories to detect A3G, the reason previous reports were unable to detect A3G in LNCaP cells may be due to the sensitivity of the antibody used in their western analysis. In addition, it is possible that the epitopes of endogenous A3G in prostate epithelial cells are not recognized by the anti-ApoC17. It is important to point out that scanning for m/z peptides from our mass spectrometry data revealed presence of peptides mapped to A3B, A3D and A3F. This is not surprising given that A3B, A3D and A3F have molecular weights similar to A3G. However, the molecular weights of A3A, A3C and A3H (~23 kDa) are substantially lower than A3G and detection of A3A/A3C/A3H in the A3G band are highly unlikely.

**Figure 3:**
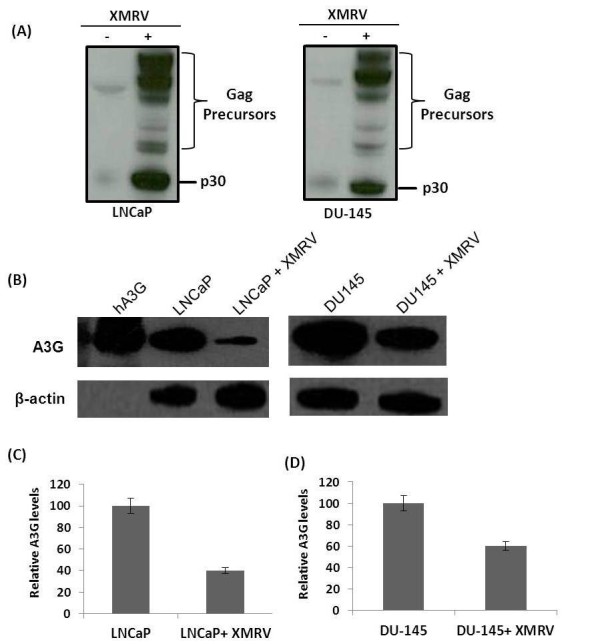
**XMRV induced downregulation of A3G in prostate cancer cells We used culture supernatant from chronically infected LNCaP cells with XMRV as the source of infectious XMRV**. Virus infections were performed using cells plated 1 day before infection. Cells were at 50% confluency at the time of infection. On the day of infection, fresh media containing 5 μg/ml polybrene was added to the cells and virus was layered on the cells and incubated for 6 h to allow virus adsorption. Cells were then washed once with PBS, and fresh media containing FBS were added. After 1-2 weeks, XMRV infection was confirmed by detecting XMRV p30 protein by using goat polyclonal anti-Rauscher MLV p30 Gag in uninfected (-) and XMRV infected (+) LNCaP cells (**A**) and DU-145 cells (**B**). (**C**) A3G expression was determined by the protocol described in Figure 1 using anti-ApoC29. (**D**) Densitometry of A3G expression as determined by three independent experiments.

Given that XMRV replicates efficiently in LNCaP and DU-145 cells [3], we examined the effect of XMRV infection on the A3G levels in these cells. We used culture supernatants from chronically infected LNCaP cells with XMRV as the source of virus. We infected LNCaP and DU-145 cells and confirmed infection by detecting XMRV p30 in these cells. We used goat polyclonal anti-Rauscher MLV p30 Gag (a gift from Dr. Sandra Ruscetti, NCI-Frederick) that reacts with XMRV p30 and its precursor Gag in our experiment (Figure 3A). Subsequently, we analyzed A3G expression in these cells by western blot analysis. Intriguingly, XMRV infection resulted in a substantial downregulation of A3G expression in both of these cell types (Figure 3B). Our densitometry analysis revealed that A3G was downregulated almost 60% in infected LNCaP cells whereas the downregulation was ~ 40% in infected DU145 cells (Figure 3C). It is important to point out that the virus used to infect these cells were produced from the LNCaP cells that express A3G. It has been shown that A3G can be efficiently packed into XMRV virions [25]. Paportka et al. have reported that XMRV produced from PBMCs with high levels of A3G proteins are infection competent and can infect new cells [17]. Since we detected robust XMRV p30 bands in our infection experiments (Figure 3B), our data suggest XMRV produced from A3G containing cells have the ability to productively infect and replicate in target cells. Therefore our findings challenge the notion that the prostate epithelial cell lines were able to produce infectious XMRV because these cells do not express A3G. Given that our results demonstrate downregulation of A3G in XMRV infected prostate cancer cells, we believe XMRV has the ability to counteract A3G antiviral function in prostate cancer cells. These results are highly significant given that XMRV is a simple retrovirus and does not encode accessory proteins that are known to degrade A3G.

In the absence of Vif-like accessory proteins, retroviruses such as Human T cell lymphoma virus (HTLV) and Murine leukemia virus (MLV) have developed alternative mechanisms to evade host restriction by A3 proteins. A motif of HTLV nucleocapsid (NC) prevents packaging of A3G into the virion [20]. Therefore exclusion of A3G has been proposed to be a common mechanism for Vif-deficient retroviruses to counteract A3G restriction. MLV virions have also been reported to exclude mA3 [21]. XMRV has been demonstrated to package A3G [16], therefore a role for exclusion mechanism is unlikely. XMRV produced from LNCaP cells show signatures of hypermutation that are characteristics of A3F [15,17]. Therefore, it is plausible XMRV is somehow resistant to A3G restriction in these cell types. XMRV may achieve this either by downregulating A3G levels or by evading A3G restriction. Given that MLV has been reported to inactivate mA3 by viral protease [22], a similar mechanism for XMRV cannot be ruled out. Furthermore, certain polymorphic alleles of A3G have been reported to increase the susceptibility to HIV infection [23]. Therefore, we are investigating whether A3G produced from prostate epithelial cells have mutations that can be assigned for cell specific susceptibility to XMRV infection. The other mechanism that could possibly explain our results is that A3G remains as high-molecular-mass (HMM) ribonucleoprotein complex in prostate epithelial cells. It has been reported that A3G in resting CD4+ cells and monocytes are predominantly in its low-molecular-mass (LMM) active form making these cells refractory to HIV-1 infection [24,25]. Conversion of LMM to the inactive HMM complex, when the CD4+ cells are activated or monocytes are differentiated into macrophages, makes these cells prone to HIV infection. Since HMM forms of A3G are reported to be enzymatically inactive, if A3G remains in HMM complex in prostate epithelial cells, it may not be able to restrict XMRV replication.

In summary, this report demonstrates the presence of A3G in prostate epithelial cell lines (LNCaP and DU145) that support efficient XMRV replication. Since XMRV packages A3G in its virions and lacks Vif-like accessory proteins, our findings on XMRV-induced downregulation of A3G may represent a new pathway by which retroviruses counteract antiviral effects of A3 proteins in human cells. Our data warrants further studies to decipher the mechanism by which XMRV may counteract restriction by A3 proteins.

## Competing interests

The authors declare that they have no competing interests.

## Authors' contributions

JP, CM, AD, BL, and CD conceived the ideas, and designed the experiments. AD, CM and JP carried out all the experiments and participated in the discussion of the data. AD and SP carried our Mass spec analysis. CD coordinated the study, and wrote the manuscript. All authors read and approved the final manuscript.
